# The effects of warm water immersion on blood pressure, heart rate and heart rate variability in people with chronic fatigue syndrome

**DOI:** 10.4102/sajp.v74i1.442

**Published:** 2018-08-28

**Authors:** Romy Parker, Zeenath Higgins, Zandiswa N.P. Mlombile, Michaela J. Mohr, Tarryn L. Wagner

**Affiliations:** 1 Department of Anaesthesia and Perioperative Medicine, University of Cape Town, South Africa; 2 Department of Health and Rehabilitation Sciences, University of Cape Town, South Africa

## Abstract

**Background:**

Chronic fatigue syndrome (CFS) is a central sensitisation syndrome with abnormalities in autonomic regulation of blood pressure (BP), heart rate (HR) and heart rate variability (HRV). Prior to exploring the effects of hydrotherapy as a treatment for this population, changes in BP, HR and HRV during warm water immersion need to be established.

**Objectives:**

The study aimed to determine the effects of warm water immersion on BP, HR and HRV in adults with CFS compared to matched-pair healthy adults.

**Method:**

A quasi-experimental, single-blinded study design was used with nine CFS participants and nine matched controls. Participants’ BP, HR and HRV were measured before, after 5 minutes and post warm water immersion at the depth of the fourth intercostal space, using the Ithlete® System and Dräger BP monitor.

**Results:**

There was a significant difference between groups in HRV prior to immersion (control group: 73 [55–74] vs. chronic fatigue syndrome group: 63 [50–70]; *p* = 0.04). There was no difference in HRV post-immersion. A significant difference in HR after immersion was recorded with the control group having a lower HR than those with CFS (78 [60–86] vs. 86 [65–112]; *p* = 0.03). The low HRV present in the CFS group prior to immersion suggests autonomic dysregulation. Individuals with CFS may have reduced vagal nerve activation post-immersion. During immersion, HRV of the CFS participants improved similar to that of the healthy controls.

**Conclusion:**

Prior to immersion, differences were present in the HRV of the participants with CFS compared to healthy controls. These differences were no longer present post-immersion.

**Clinical implications:**

Warm water immersion appears safe and may be beneficial in the management of individuals with CFS.

## Introduction

Chronic fatigue syndrome (CFS) is a complex disorder, which exhibits variability in both symptoms and aetiologies (N.I.C.E. 2007; White et al. 2011). Currently, its worldwide prevalence is between 0.2% and 2.6% (White et al. 2011). In South Africa, no studies have established its prevalence; however, anecdotal evidence estimates it to be between 0.5% and 1.5% (Potocnik 2016). The terms ‘CFS’ and ‘myalgic encephalomyelitis’ are often used interchangeably; in this article, we will only use the term ‘CFS’ (White et al. 2011).

Chronic fatigue syndrome is characterised by chronic debilitating fatigue during minimal exertion, followed by a prolonged period of recovery (Carruthers et al. 2011; White et al. 2011). It comprises a range of autonomic and neurocognitive symptoms that vary in both frequency and intensity (Carruthers et al. 2011; Meeus & Nijs 2007; N.I.C.E. 2007) and often results in reduced functional capacity (Chambers et al. 2006). The mechanism responsible for the generalised hypersensitivity as well as pain and fatigue in the absence of tissue damage experienced by individuals with CFS is known as central sensitisation (Bourke, Langford & White 2015; Meeus & Nijs 2007; Nijs et al. 2011, 2012). Individuals with CFS exhibit sympathetic hyperactivation as well as parasympathetic dysregulation. This leads to an inability to respond appropriately to stressors, resulting in symptoms such as fatigue, stiffness, sensitive tender points, exercise intolerance and sleeping difficulties. Because of the effects of central sensitisation on the autonomic nervous system (ANS), blood pressure (BP), heart rate (HR) and heart rate variability (HRV) are thus affected (Meeus et al. 2013).

The 2011 International Consensus Criteria are the most recent and widely used method of diagnosing an individual with CFS, be it mild, moderate or severe (Carruthers et al. 2011). Currently no known medical treatment for CFS is available (N.I.C.E. 2007); however, management approaches used in other central sensitisation syndromes, such as fibromyalgia, are commonly implemented and include psychological, physical and pharmacological interventions (Edmonds, McGuire & Price 2013). It has been suggested that exercise is the most effective treatment for CFS (N.I.C.E. 2007), with graded exercise therapy (GET) providing the greatest success in reducing fatigue and improving physical function (Chambers et al. 2006; White et al. 2011). Physiotherapists often use hydrotherapy as part of a graded exercise regimen in the treatment of CFS; however, limited evidence exists regarding its use, more specifically its effects on HR, HRV and BP. Prior to investigating the effects of immersion on BP, HR and HRV in people with CFS, a review of the effects of immersion on healthy individuals is indicated.

According to Bates and Hanson (1996), healthy individuals immersed at the level of the chest or higher, experience an increase in external pressure on the circulatory system as a consequence of hydrostatic pressure (for every 10 m of immersion, pressure increases by 1 atm). The increase in external pressure promotes the displacement of blood in a cephalad direction, towards the great vessels of the heart, leading to an increase in central blood volume (Bates & Hanson 1996). Therefore, in response to immersion, an elevation in arterial BP, pulmonary arterial pressure, cardiac output and cardiac volume occurs (Cole & Becker 2004). The above-mentioned response to immersion is largely influenced by activity of the ANS, which is responsible for maintaining diastolic BP through control of smooth muscle contraction in the walls of the blood vessels. HRV is defined as the variation in time between consecutive heartbeats. In a healthy individual, there is a large variation in HRV as a consequence of the interaction between the sympathetic and parasympathetic systems of the ANS. During immersion, healthy individuals experience compensatory changes in both HR and HRV. Analysis of HRV therefore provides an insight into the activity of the ANS (Beavers 2013).

To our knowledge, no studies assessing the effects of immersion on cardiac autonomic control in people with CFS exist. Zamunér et al. (2015) investigated changes in HRV in fibromyalgia, also a central sensitisation syndrome, during hydrotherapy and found that participants presented with non-linear dynamics in HRV. This suggests that the integrity of cardiac autonomic regulation in individuals with fibromyalgia is compromised compared to healthy subjects. However, the study was not blinded, indicating that a bias may be present, and did not focus on BP responses during immersion (Zamunér et al. 2015).

Chronic fatigue syndrome is a central sensitisation condition with possible abnormalities in the autonomic regulation of BP, HR and HRV (N.I.C.E. 2007). Given that in healthy individuals immersion has been found to influence BP, HR and HRV, it is worth exploring the effects of immersion on BP, HR and HRV in people with CFS. This information will then be used to inform future studies exploring the effects of hydrotherapy in people with CFS.

### Aims and objectives

The study aimed to determine the effects of warm water immersion on BP, HR and HRV in adults with CFS compared to matched-pair healthy adults.

The specific objectives of the study were to compare volunteers from the South African (SA) CFS support group, diagnosed according to the 2011 International Consensus Criteria, to matched healthy controls in order to determine if differences in BP, HR and HRV within and between groups was present before, during and after immersion.

## Method

This study followed a pre-test, post-test–equivalent control group quasi-experimental, single-blinded design. The investigators were blinded during data analysis to limit bias.

Individuals with CFS were recruited via an advertisement on the SA CFS support group website. Matched controls, from the same suburbs in which the CFS participants resided, were recruited via an advertisement in newspapers. Potential participants were then contacted via email and the study was explained. Those who agreed to participate were screened for inclusion and exclusion criteria and demographic information was obtained.

In order to participate in the study, the CFS participants needed to have at least a 6-month history of CFS and meet the 2011 International Consensus Criteria for CFS as diagnosed by a personal physician; be between the ages of 18 and 60 years and be willing to be immersed in a hydrotherapy pool at the depth of the fourth intercostal space. Matched controls needed to be between the ages of 18 and 60 years, be willing to be immersed in a hydrotherapy pool at the depth of the fourth intercostal space, reside in the same suburb as the recruited participants with CFS and have a stable health status (no history of illness, injury or co-morbidities) for the past 6 months. The controls must not have been diagnosed with CFS nor any other central sensitisation syndrome (i.e. fibromyalgia) and have a body mass index (BMI) similar to that of the control to which they were matched.

Participants and matched controls were excluded from the study if they were currently taking beta-blockers, had been diagnosed with clinical hypertension or presented with contraindications to hydrotherapy.

A sample of convenience was used as challenges recruiting chronically ill participants, with both limited time and availability, were faced. A minimum sample size was determined based on data from Prinsloo et al. (2011), who conducted a study on HRV biofeedback in people during laboratory-induced cognitive stress. The authors stated that although their sample size was small (18 participants, 9 in each group), it was adequate as the main outcome provided significant results, therefore ensuring sufficient power (0.95) (Prinsloo et al. 2011). Based on these data, a minimum of 18 participants was therefore required for the study (*n* = 18).

### Measurement instruments

Blood pressure was measured using the Dräger Infinity M540 patient monitoring system that records BP non-invasively, via a static cuff with an accuracy of ±3 mmHg (Dräger 2016). This monitor uses an oscillometric method with a step deflation technique, decreasing bias caused by human error and is therefore less vulnerable to external interference (Pérgola, White & Graves 2007; Pickering et al. 2005). The M540 monitor has been found to produce both valid and reliable BP recordings (Martin et al. 2016).

The primary instruments used to measure HR and HRV include an electrocardiogram (ECG), blood volume pulse (BVP) and online HR monitors (Combatalade 2010). In this study, a BVP monitor was used. A BVP shines infrared light through the finger and measures the reflection of light occurring within the vessel, which is then converted into HR and HRV data (Combatalade 2010). Ithlete®, a smartphone application, determines HR and HRV using an Ithlete® BVP Finger Sensor, which transmits a signal from a matched infrared light–emitting diode and photodiode embedded in a silicone finger clip. The Ithlete® Finger Sensor was connected to an iPhone, which accurately recorded HR and HRV (Heathers 2013). The Ithlete® application has been found to be a valid and reliable measure of HR and HRV over a period of 55 s when compared to the gold standard of an ECG (Flatt & Esco 2013).

**Demographic information**: A self-designed questionnaire was used to gain demographic information from the participants, as well as to ensure inclusion and exclusion criteria mentioned above. Demographic information included age, occupation, language, suburb of residence and race.

**Chronic fatigue syndrome symptom list**: The CFS symptom list created by Nijs, Aerts, and De Meirleir (2006) was used to identify the main symptoms experienced by individuals with CFS. These data were used to assess the severity of CFS symptoms as well as to ensure no controls suffered from undiagnosed CFS (Nijs et al. 2006).

**Weight, height and body mass index:** These parameters were assessed using an Adam MDW-250L, which consists of a calibrated scale and stadiometer. Papandreou et al. (2015) and Al-Rethaiaa, Fahmy and Al-Shwaiyat (2010) found that both the scale and stadiometer produced valid and reliable data. Each participant’s height (m) and weight (kg) values were used to calculate BMI:$BMI=\frac{kg}{m^{2}}$ (Al-Rethaiaa et al. 2010; Papandreou et al. 2015).

### Procedure

With permission, an advertisement for the study was placed on the SA CFS support group website. Potential CFS participants contacted the researchers and were recruited via email, which briefly explained the study and screened for inclusion and exclusion criteria. To standardise BP measurements, all participants were asked to attend a session at Groote-Schuur Hospital physiotherapy department between 08:00 and 13:00.

Before participants arrived, the temperature of the pool was checked to be 35 °C to 37 °C (Beavers 2013). Warm water immersion is defined as immersion in water of a temperature ≥ 36 °C. Immersion in water of this temperature raises the core body temperature. On arrival, the authors explained the study, the contents of the information sheet and consent form. The participants were familiarised with the procedure, including orientation to data collection equipment and the hydrotherapy pool and given the opportunity to withdraw. If participants consented, consent forms were completed. Inclusion and exclusion criteria were then re-assessed and the CFS symptom list was completed. Height and weight were measured in barefoot standing using an Adam MDW-250L. The height of the fourth intercostal space (Cook, Roberts & Weinhaus 2009) was measured using a metal ruler and the depth of the pool adjusted to this height using the hydraulic floor mechanism. All measurements were taken by the same authors throughout (HR and HRV measured by T.W., BP measured by M.M.).

Participants changed into swimwear and sat on a chair in a relaxed posture for 5 min prior to measurement of BP, HR and HRV using an Ithlete® finger sensor linked to the Ithlete® application and a Dräger Infinity M540 BP monitor, respectively. Thereafter participants stood and showered in lukewarm water (≤ 35 °C) for less than 1 min prior to entering the pool. An iPhone 6 device was used to measure 5 min, in which participants stood immersed, with arms supported at the side of the pool (Petrie et al. 1986).

At 5 min BP, HR and HRV were measured while the participant remained standing in the pool using the same procedure described above. Participants were then asked to exit the pool and return to the initial seated posture for 5 min, after which BP, HR and HRV were measured in the same manner as initially. Once the third reading was obtained, monitors were removed. Participants then showered and dressed prior to departure. Lastly, reimbursement for transport was given and symptom management was discussed. All data were obtained individually and documented. Once data had been collected from the CFS participants, advertisements were placed in community newspapers and matched controls who responded via email were recruited. The same procedures were followed for the matched controls. Once the study was complete, the key role players were contacted via email and the findings explained.

### Statistical analysis

Because of the small number of participants (< 10 in each group), sample of convenience and non-parametric data, non-parametric approaches were used to describe and analyse the data using STATISTICA 13.0. Results are presented as median and range with significance accepted as *p* ≤ 0.05. Differences between groups for participant characteristics were tested using the Mann–Whitney *U* test. Differences within groups for changes in BP, HR and HRV before, during and after immersion were tested using the Friedman’s test with subsequent analysis using the Wilcoxon matched-pairs test. Where significant differences between groups were found in the Mann–Whitney *U* or the Wilcoxon matched-pairs tests, effect sizes were calculated as *η*^2^ and converted to Cohen’s *d*.

### Ethical considerations

Ethical approval was obtained from the University of Cape Town, Faculty of Health Sciences Human Research Ethics Committee (HREC Ref: 077/2016).

## Results

### Demographic information and chronic fatigue syndrome symptom list

The characteristics of the participants are presented in Table 1. There were no differences between participants in the control and CFS groups, in terms of age, height, weight and BMI (Table 2). In addition, the CFS group had significantly worse scores (48.4/190 [3.9–115.2]) than the control group (19.2/190 [0.8–23.2]) on the CFS symptom list (*U* = 10; *p* < 0.01; *d* = 1.6).

**TABLE 1:** Participant characteristics.

Participant code	CFS or control	Age (y)	Height (m)	Weight (kg)	BMI (kg/m^2^)	CFS symptom list (×/190)
A1	Control	18	1.750	69.1	22.56	13.7
B1	CFS	18	1.650	47.0	17.26	91.2
A2	Control	22	1.770	80.5	25.70	23.2
B2	CFS	22	1.780	90.7	28.63	115.2
A3	Control	23	1.680	74.2	26.29	19.2
B3	CFS	21	1.735	112.2	37.27	3.9
A4	Control	23	1.750	65.8	21.49	4.3
B4	CFS	22	1.750	70.2	22.92	48.4
A5	Control	23	1.690	71.8	25.14	19.2
B5	CFS	25	1.660	77.7	28.20	96.5
A6	Control	30	1.600	75.5	29.49	20.0
B6	CFS	32	1.750	73.1	23.87	34.9
A7	Control	56	1.760	89.7	28.96	23.2
B7	CFS	58	1.720	91.0	30.76	56.9
A8	Control	40	1.665	82.4	29.72	11.2
B8	CFS	42	1.900	70.2	19.45	46.6
A9	Control	31	1.580	67.4	27.00	0.8
B9	CFS	30	1.660	80.8	29.32	22.1

CFS, Chronic fatigue syndrome; BMI, body mass index.

**TABLE 2:** Demographic characteristics of the participants (*N* = 18).

Variable	Controls (*n* = 9)	CFS Participants (*n* = 9)	Statistical test	
	median (range)	median (range)	Mann–Whitney *U*	*p*
Age (y)	23 (18–56)	25 (18–58)	39	0.93
Height (m)	1.69 (1.58–1.77)	1.74 (1.65–1.9)	35	0.66
Weight (kg)	74.2 (65.8–89.7)	77.7 (47–112.2)	31	0.43
BMI (kg/m^2^)	26.29 (21.49–29.72)	28.19 (17.26–37.27)	40	1.00

CFS, Chronic fatigue syndrome; BMI, body mass index; *n*, number.

### Systolic blood pressure

**FIGURE 1:**
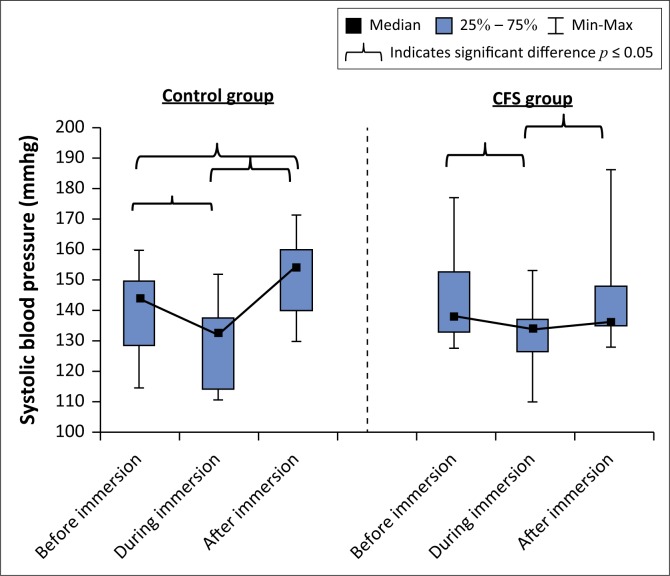
Box and whisker plot showing changes in systolic blood pressure before, during and after immersion of the control and chronic fatigue syndrome groups.

Within-group analysis showed significant changes in systolic BP over time in both groups (Figure 1). In the control group, significant differences in systolic BP were present between all measures. However, in the CFS group, no significant differences in systolic BP were recorded from before immersion to after immersion. No differences in systolic BP between the control group and CFS group before, during and after immersion were found (Table 3).

**TABLE 3:** Heart rate variability of the control and chronic fatigue syndrome groups before, during and after immersion (*N* = 18).

Variable	Control group (*n* = 9)	CFS group (*n* = 9)	Statistical test		
	Median (Range)	Median (Range)	Mann–Whitney *U*	*p*	Cohen’s *d*
HRV before immersion	73 (55–74)	63 (50–70)	16.50	0.04*	1.2
HRV during immersion	68 (65–82)	67 (49–73)	23.50	0.15	-
HRV after immersion	71 (57–93)	69 (50–72)	22.00	0.11	-

CFS, Chronic fatigue syndrome; HRV, heart rate variability.

* , Indicates significance with *p* ≤ 0.05.

### Diastolic blood pressure

**FIGURE 2:**
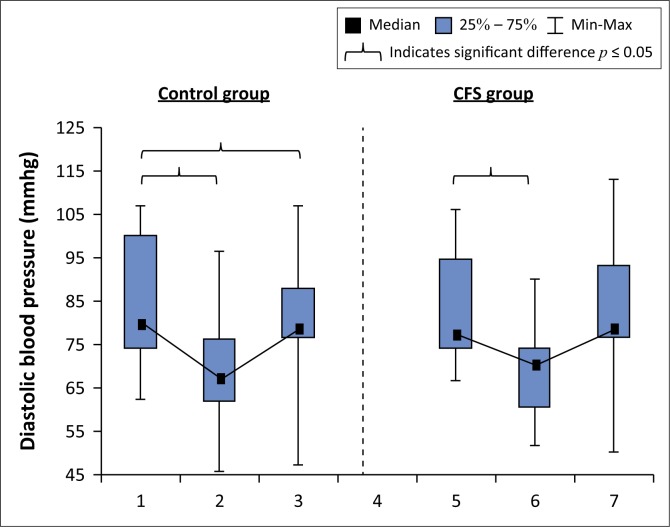
Box and whisker plot showing changes in diastolic blood pressure before, during and after immersion of the control and chronic fatigue syndrome groups.

Within-group analysis showed there were significant changes in diastolic BP over time in both groups (Figure 2). There were no differences in diastolic BP between the control group and CFS group before, during and after immersion (Table 3).

### Heart rate

**FIGURE 3:**
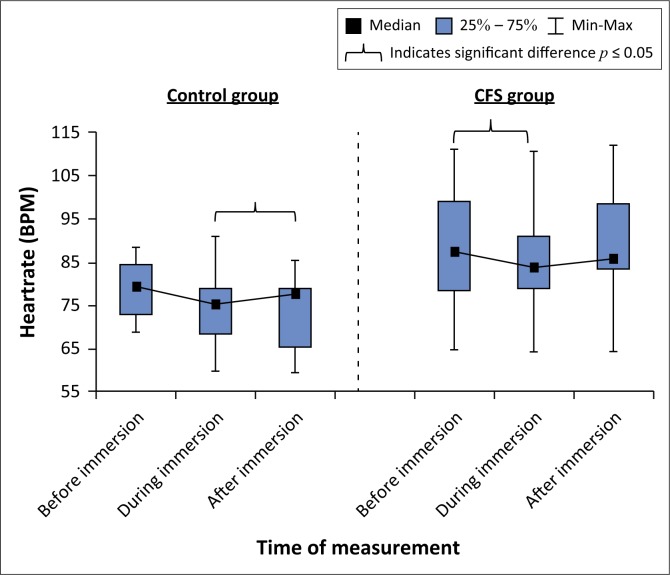
Box and whisker plot showing changes in heart rate before, during and after immersion of the control and chronic fatigue syndrome groups.

Within-group analysis showed there were significant changes in HR over time in the control (χ^2^ = 6.23; *p* = 0.04) and CFS (χ^2^ = 6.34; *p* = 0.04) groups (Figure 3). There were no differences in HR between the groups before and during immersion. However, after immersion the control group had a lower HR than the CFS group (78 [60–86] vs. 86 [65–112]; *U* = 15, *p* = 0.03; *d* = 1.3).

### Heart rate variability

**FIGURE 4:**
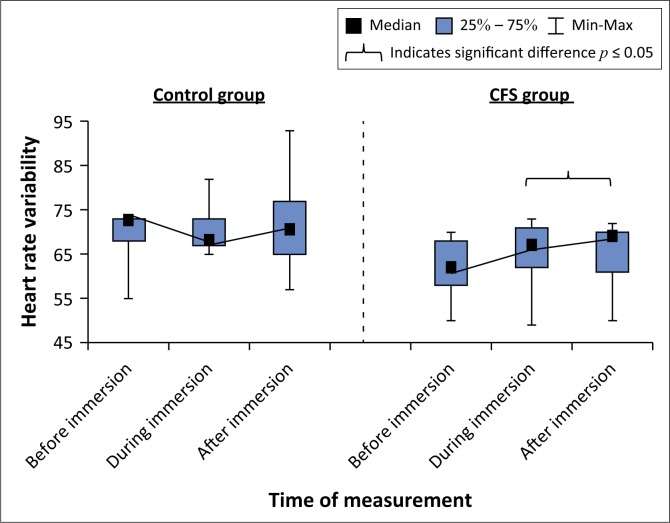
Box and whisker plot showing changes in heart rate variability before, during and after immersion for control and chronic fatigue syndrome groups.

Within-group analysis showed there were no significant changes in HRV over time in either groups (Figure 4). There was a significant difference between groups in HRV before immersion with the HRV of the control group being higher than the CFS group (73 [55–74] vs. 63 [50–70]; *U* = 16.50, *p* = 0.04; *d* = 1.2). However, there were no differences in HRV between the control group and CFS group during and after immersion (Table 3).

## Discussion

This study aimed to determine the effects of warm water immersion on BP, HR and HRV in individuals with CFS, as well as to determine whether significant differences in BP, HR and HRV were present between the CFS and control groups. While the groups were well matched in terms of demographic characteristics of suburb they lived in, gender and BMI, differences in height may have influenced the results. The results of the CFS symptom list showed significant differences between the CFS and control groups, ensuring the control group did not suffer from undiagnosed CFS.

Both groups demonstrated a change in systolic and diastolic BP to immersion as expected when exposed to the increase in external pressure on the circulatory system (Bates & Hanson 1996). There were no significant differences between the groups for BP before, after 5 min of immersion and post-immersion. However, the results from the Wilcoxon matched-pairs test did indicate that while the control group showed an overall significant change in systolic BP from before to post-immersion, the CFS group had no significant change in systolic BP during this time. In Figure 1 it can be observed that the control group showed the expected significant decrease in systolic BP when immersed, followed by a significant increase after 5 min of immersion (Bates & Hanson 1996). The CFS group on the other hand had a decrease in systolic BP after 5 min of immersion followed by an increase post-immersion.

Cook et al. (2012) investigated responses to exercise in patients with CFS and fibromyalgia and found that individuals with central sensitisation display ANS dysfunctions, leading to chronic blood flow abnormalities. These autonomic dysfunctions may be responsible for the slow BP responses observed during immersion as well as the recovery from immersion of the CFS participants recorded in our study (Cook et al. 2012). Although some differences between the groups in our study in the BP response to immersion can be identified, no conclusions regarding the effects of immersion on systolic and diastolic BP can be made because of the lack of significant differences between the groups, the distribution of the data and the limitations of the study design. Nevertheless, further study using a larger sample size allowing more rigorous data analysis appears warranted.

The results also suggest that the individuals with CFS showed no significant difference in HR before immersion when compared to healthy controls. However, post-immersion, a significant difference between the two groups was identified. This suggests that HR responses to immersion and recovery from immersion in the CFS group may be impaired. van Oosterwijck et al. (2015) investigated impairments in the ANS at rest, during and post-exercise in patients with CFS and found that reduced parasympathetic reactivation was present during recovery from exercise, as recorded by electrophysiological measures. Their study also found that after exercise the control groups’ HR quickly recovered to its pre-exercise value, while the CFS participants had a significantly longer recovery time, with the 10-min period being inadequate for the return of HR to original levels (van Oosterwijck et al. 2015). Although no exercise was performed during our study, a clear parallel can be identified between the two studies, with the CFS group’s decreased parasympathetic reactivation causing HR recovery to be reduced when compared to the controls. Therefore, it appears that individuals with CFS have reduced parasympathetic nervous system activation post-immersion when compared to healthy controls. It also appears that immersion has no significant direct effect on HR in individuals with CFS.

The most noteworthy finding of our study was that the individuals with CFS showed a significant difference in HRV before immersion when compared to the healthy controls, which reduced to no difference post-immersion. The difference in HRV prior to immersion supports the report of Meeus et al. (2013) of abnormalities in HRV in individuals with CFS when compared to healthy controls, because of hyperactivity of the sympathetic nervous system and reduced activation of the parasympathetic nervous system, suggestive of underlying autonomic dysregulation. These results also emulate those of Zamunér et al. (2015) on HRV in fibromyalgia, who established that participants initially presented with non-linear dynamics in HRV, suggestive of compromised integrity of cardiac autonomic modulation in individuals with central sensitisation (Zamunér et al. 2015). The results of our study may suggest that immersion positively affects HRV in people with CFS. This is similar to the results of Zamunér et al. (2015), who found that a hydrotherapy program was effective in normalising the cardiac sympathetic modulation and complexity of HRV as well as improving cardiac autonomic adjustments to orthostatic pressure in fibromyalgia, that is, hydrotherapy appears to normalise the functioning of the ANS in people with a central sensitisation syndrome (Zamunér et al. 2015).

### Limitations

Because of the restricted availability of participants, the study made use of a small sample requiring the use of non-parametric analysis. This limits both the power and the generalisability of the study. In addition, the distribution of the data limits interpretation of statistical differences recorded between groups. Data collection also took place over a 5 hour period and the impact of circadian rhythms may have influenced the BP results obtained during the study, introducing greater variability in the results. However, given the limited research on the pathology and treatment of CFS, the results are worthy of consideration despite the limitations. To improve the reliability and generalisability of the results, further research needs to be conducted using a larger sample size with greater control of circadian variability and matching on more socio-demographic variables. In addition, the use of a time series design with each participant observed multiple times would provide more meaningful data on the nature of the responses to immersion. However, it must be noted that the use of a time series design will need to be carefully considered for people with CFS who suffer with post-exertional neuroimmune exhaustion, which may require extended breaks (more than a week) between data collection points.

## Conclusions

Chronic fatigue syndrome is a central sensitisation syndrome that presents with abnormalities in autonomic regulation of BP, HR and HRV. Limited research regarding the effects of immersion on individuals with CFS exists and therefore a study was conducted to establish the effects of immersion on BP, HR and HRV. The study found that prior to immersion; differences were present in HRV of the participants with CFS when compared to healthy controls. These differences were no longer present post-immersion. This finding may provide some justification for the use of immersion as a form of GET in the management of individuals with CFS, as warm water immersion may be a beneficial regulator of the ANS in this patient group. It may also be proposed that individuals with CFS have reduced parasympathetic nervous system activation post-immersion when compared to healthy controls, leading to a reduction in HR recovery post-immersion. Lastly, no significant detrimental effect on HR or BP in individuals with CFS occurred in the participants during or post-immersion, suggesting that immersion in warm water, as used in hydrotherapy, appears safe for this patient group. This preliminary study only investigated participants standing immersed for 5 min and further research appears warranted to explore whether hydrotherapy exercise is effective in the management of individuals with CFS.
